# Hospital and Institutional News

**Published:** 1919-11-15

**Authors:** 


### HOSPITAL AND INSTITUTIONAL NEWS.

### MORE WORDS ON THE HOSPITAL CRISIS.

It is with very real satisfaction we are able to report the facts stated in the leading article on page 131 of the present issue, and many things are tending to demonstrate, that, the faint hearts, who have committed themselves to the statement that the voluntary hospitals cannot continue to pay their way, show a lack of appreciation of the hold which the voluntary system rightly has on British people. Indeed those who have so readily shown a disposition to throw up the sponge are losing justification for their fears in increasing volume every week. The change in the financial policy intimated by the Government in the House of Commons at the end of October has materially improved the outlook of the voluntary hospitals, and the contributions to these institutions are showing a marked tendency to improve in consequence.

### PROGRESSIVE EFFORTS THROUGHOUT THE COUNTRY.

Last week we published a stirring appeal to the people of Glasgow, one of the most prosperous cities during the war, to raise £150,000 and establish, without delay, an interesting and lasting memorial to Lord Lister. Immediate action has been taken. The Royal Infirmary Board understands very well the great desirability of doing all the work in Glasgow foreshadowed on pages 119/121 in The Hospital for the 8th inst., and an influential committee already has been appointed to deal with this important and pressing matter. As evidence that this spirit of earnest, purposeful endeavour is awakening among the voluntary hospital managers up and down the country, we have the pleasure to publish this week an extension scheme involving an expenditure of £50,000 in connection with the Radcliffe Infirmary and County Hospital, Oxford. Already an appeal fund has been opened and a sum approaching £20,000 has been promised (see page 141). This scheme confers the greatest credit on the Rev. G. B. Cronshaw, the able treasurer, and the committee and medical staff of the Radcliffe Infirmary. We hope that Oxford people, who have now joined Glasgow in the race to place Oxford in the forefront of successful and well-financed voluntary hospitals, will run a neck-and-neck race as to who shall first succeed in raising the money required by January 30, 1920, or earlier. Another great county hospital, the Leicester Royal Infirmary, which did splendid work during the war, and is continuing it with commendable vigour and energy, has received a gift of £20,000 from the executors of the late Mr. Thomas G. Langham, whose family have been long and prominently associated with agricultural interests in the county. The gift is free from restrictions and conditions, and the executors suggest that the money should be used in the building of an orthopædic ward and an isolation hospital. The appeal from St. Bartholomew's Hospital is proceeding satisfactorily, and we have reason to believe that the change in the financial policy of the Government will materially improve the probabilities that the £500,000 needed will be forthcoming in due course.

### £285,310 DUE TO LONDON HOSPITALS.

We last week showed that the sum expended by 109 London hospitals, alone, in a single year, was £285,310 more than the grant received from the Government authorities. We have pleasure in expressing the view, which rests on good authority, that if an application is made by these hospitals to the Government for this large sum (£285,000) there is good reason to believe it will be paid to them with full recognition of the services they have rendered to the country and the nation.

### HARD WORK CAN SOLVE FINANCIAL CRISIS.

Here, then, is noteworthy testimony to the contention that, providing sufficient energy and hard work are brought to bear, not a single voluntary hospital of importance need fear the present financial stress, which affects not only institutions, but the majority of the people in Great Britain. They still have at heart the best interests of the voluntary hospitals, and will not fail to the utmost extent of their means to help the managers of the best institutions to weather successfully the present financial crisis. We shall be glad to have an early intimation, with particulars of each hospital which decides boldly to face the crisis by providing that no effort shall be wanting on the part of its managers to secure all the financial aid which they may need to weather the storm and keep their institution at the highest point of efficiency. The first leading article this week, pages 131 and 132, should be read in this connection.

### COLONIAL MEDICAL SERVICES.

The Secretary of State for the Colonies has appointed a Committee, under the Chairmanship of Sir W. Egerton, late Governor of British Guiana, to consider the position of the medical services of the various colonies and dependencies. News from many quarters shows the urgent need of revision and reconstruction, both with a view to maintaining and increasing the supply of candidates and to securing contentment and resulting maximum efficiency in the services themselves. The principle of assimilating the medical service of neighbouring colonies is to be brought under consideration with a view to general extension. Most is publicly heard of our outposts of Empire, but relatively little of that all-important factor their medical provision, and there is no doubt that, in view of modern world tendencies, extensive changes and developments are necessary. We wish this committee all success in the rapid solution of the host of problems they must inevitably encounter.

### VACCINATION FEES INCREASED.

We learn that the Minister of Health has now decided to issue an order providing an increase to 5s. for the minimum fee prescribed for primary vaccination performed at the homes of persons vaccinated. This new order will not come into force until

December 1, and will not, of course, affect public vaccinators, who are already receiving this new minimum or a higher fee. The increase is at least a recognition of the importance of what may be and generally is, under favourable conditions, a very simple operation, but one which requires careful technique and which, particularly in the case of very young children in the face of a threatened smallpox outbreak, may be associated with at least as much responsibility as attaches to many much more highly rewarded operations.

### SALE OF A HOSPITAL SITE.

AN announceme t has been made to the effect that the site upon which the Hospital for Diseases of the Throat stands, in Golden Square, will shortly be in the market. The site is freehold, is not far from Piccadilly Circus, and has no doubt enormously appreciated in value during recent years; especially as the property, besides Nos. 32 and 33 Golden Square, comprises also 1, 2, 3, and 4 Upper John Street, and 22 and 24 Beak Street. The statement that has appeared says that the rapid expansion of the work of the hospital renders its removal to a larger site imperative. What site the committee have in mind has not transpired; and until an option or a provisional contract has been signed it is, of course, not advisable or desirable, from the business point of view, that it should be made public. Possibly the policy of amalgamation of throat and nose hospitals, which the King Edward VII. Fund executive is known to favour, is about to materialise. At all events the decision as to the future of the Golden Square Hospital will be awaited with considerable interest in London institutional circles. It is to be hoped that a large sum will be realised by the sale of the old site: the Throat Hospital is lucky to be the owner of its own freehold, and should find itself well situated after a successful sale, either to emigrate or to amalgamate upon favourable terms.

### THE END OF THE ROAD.

THE cinematograph for purposes of propaganda has been approved by the Ministry of Health and the National Council for Combating Venereal Diseases, but whether their approval as applied to the film, "The End of the Road," implies that it is fit for general public instruction, is another matter. It was shown privately a few days ago at a West-End Theatre, and members of the London County Council, the Press, and others, were invited to be present. It must frankly be said that the film must be cut down and considerably revised before it can be released (we think that is the technical word) in the ordinary picture-house. Some of the incidents are, to speak mildly, extremely unpleasant, but they are quite open to modification. If it is to serve its purpose—that of public instruction by popular means—it must be severely revised. When this is done the obvious sincerity will help it to focus the public mind on one of the most pressing questions of the day. The film should then do excellent work in the campaign which the National Council for Combating Venereal Disease is now carrying on.

### DEATH OF A NOTED HOSPITAL K.C.

THE death of Mr. R. H. Horton-Smith, K.C., in his eighty-eighth year, removes one who had done much in his long and useful career for London hospitals. For many years he was on the Committee of King's College Hospital, and was also a member of the College Council. In 1904 he became a member of the Committee of the Brompton Hospital for Consumption and Diseases of the Chest. He was also for many years Chairman of the Public Dispensary, Drury Lane, from which he received only a fortnight before his death an address of gratitude for his long labours in the dispensary's behalf. All this in addition to eminence at the Bar, a very prominent position and long years of work in Freemasonry, distinguished service to the Imperial Maritime League, a great interest in musical affairs, and hard work in politics! Not a bad record of all-round energy and culture. Mr. Horton-Smith always took a deep interest in medicine, and prided himself on keeping closer touch with medical science than any man not himself actually in the profession of medicine.

### ELECTION TO THE GENERAL MEDICAL COUNCIL.

PRACTITIONERS are reminded by the Registrar of the General and English Branch Councils of the General Council of Medical Education and Registration of the United Kingdom that it is probable that many addresses registered in 1914 are no longer accurate, and a practitioner who has not received a voting paper for the forthcoming election should communicate with the Registrar of the Branch Council at which he was originally registered.

### THE ROCKEFELLER INSTITUTE.

IN these days of general outcry for more active medical research, it is most satisfactory to read that a further gift of £2,000,000 has been received from its founder by the Rockefeller Institute of America. Though founded as relatively recently as 1901, this institute has become the largest endowed home of medical research in the world. The value to mankind of its already published work is too well-known to need description, but it is to be remembered that this result has been largely brought about by the magnificent gifts of nearly £6,000,000 which Mr. Rockefeller has already made. Now, even wider fields are to be dealt with—namely, medicine in relation to biology, chemistry and physics, and yet more important, the further practical study of diseases common to animals and men. Magnificent possibilities are opened to research workers acting under such financial support. Both their achieved results and their prospects should serve at least to remind us how well and deservedly may monies in this country be devoted to a similar cause.

### A CORONER'S CRITICISM.

AT a recent inquest the coroner drew attention to the wording of the printed order and summons served upon the medical man concerned in the case. This stated, *inter alia*, "You are also required to make or assist in making a *post-mortem* examination of the body, which shall comprise an examination of the viscera of the head, chest, and abdomen. The examination to be made as early as possible." In this particular instance the medical man stated he had found quite sufficient evidence of the cause for death in parts of the body other than the head, and had not made a detailed examination of the skull and brain. The coroner, therefore, found it technically necessary, before the jury could return a true cause of death, to request him to return to the mortuary to complete his work, and was to some extent justified in his most critical comments on failure in making *complete* examinations as legally required and the obvious necessity of avoiding waste of time. Being ourselves in possession of the details of this case we are, however, of the opinion that the actual medical facts do not suggest the necessity for excessive severity of comment and we would ourselves criticise the action of at least one lay paper in publishing a very incomplete account of the occurrence under a heavy-type heading of "Slovenly Doctors." The average practising doctor is, in point of fact, most infrequently called upon to make these examinations, and it is somewhat unfortunate that a local criticism, even if deserved, should become an implied indictment of the profession as a whole.

### VACANT ADMINISTRATIVE APPOINTMENTS.

We notice that the Royal Sussex County Hospital, Brighton, after a period of temporary secretaries, is advertising for a permanent officer, from which it must be gathered that Mr. Reginald B. Jay on his return from military service is not taking up his old appointment. The salary offered is £250 a year, with £100 allowance in lieu of apartments and board; candidates must be between 25 and 40 years of age, and are required to have had at least three years' training in administrative and secretarial work in a hospital of not less than 150 beds. The emoluments offered are on the low side but no doubt the post, with prospects of advancement, will be attractive to some junior officers. At the Royal Victoria Hospital, Folkestone, for a similar appointment, embracing the duties of secretary, superintendent and almoner, needless to say a wholetime post, an endeavour is being made to secure a gentleman who will accept a salary of £200 a year. No doubt it is thought that somebody with private means will come forward, who will not look upon the financial arrangement in the most critical business light and will be willing to give part of the value of his services to the hospital. At the General Hospital Birmingham, a male inquiry officer is required, and the commencing emoluments offered are £175 a year with partial board. Values in Birmingham and Folkestone are apparently not the same.

### A BETTER TYPE OF MIDWIFE.

In her quarterly report to the Durham County Council, the inspector of county midwives mentions that in many districts, even where a trained midwife has been appointed, the "handy woman" continues her activities. Generally she pleads that she was forced to act as no one else was at hand. On making inquiries, however, it is often found that the midwife could not possibly be present owing to the delay in sending for her. The patient and this officious neighbour seem to expect that the midwife will be highly pleased to find the case over when she arrives. It is most difficult to make these people realise that the midwife much prefers to conduct the confinement herself. The menace to the midwife's successful practice will only cease when the patient is educated to the vital importance of having skilled attendance at child-birth. It is, adds the inspector, gratifying to note that the improved conditions for practising midwives appear to be attracting a good type of woman to train for this work.

### MASSAGE ESTABLISHMENTS AGAIN.

Further powers are to be sought from Parliament to enable the London County Council to exercise more stringent supervision over establishments for massage and special treatment. After four years' experience the Council has found its existing powers of supervision to be insufficient to effect the suppression of what the Council's Public Control Committee calls "the serious social evil attaching to so many West-End massage and manicure establishments." Now all that is necessary is for such places to be registered when they are established, and registration cannot be reviewed unless such serious conditions arise as will enable the Council, in the event of an appeal against cancellation, to satisfy a magistrate that its action is justified. Although considerable suspicion may attach to an establishment, the Council has now no power to take action in the absence of sufficient evidence, which, it will readily be understood, is most difficult to obtain. The Council thinks the difficulty can be met by a system of annual licensing, by which all circumstances could, if necessary, come under review once a year, as is the case with employment agencies, which are licensed by the Council. As the Public Control Committee points out, annual licensing would undoubtedly prove more effective in ensuring a higher standard of conduct than at present obtains in certain establishments.

### EVIDENCE OF ADEQUATE TRAINING REQUIRED.

Cases have come to the notice of the Council of registered persons with unsuitable associates; women carrying on business for and on behalf of persons with bad characters and records; women unsuitably and immodestly attired when engaged in giving body massage to male patients; a registered person exercising no supervision over the business, being practically always absent, and leaving the management to young and unqualified assistants; and the extension of a registered manicure business to include body massage in most unsuitable premises. Difficulty has also arisen from the fact that immoral user of the premises is expressed in the statute in the present tense. Cases are continually coming to knowledge in which the accommodation and provision for treatment falls short of a reasonable professional standard, and it is very desirable, in the public interest, that the Council should be enabled to take effective action if, in its opinion, such standard is too low. In particular, it is necessary that the Council should be in a position to deal with cases in which it is not satisfied with the professional qualifications of the registered person or the assistants. In a number of establishments which are not above suspicion, the assistants possess certificates that are easily and quickly obtained after insufficient training. A definite standard of training should be insisted upon. The Council, therefore, suggests that both a licensed person and any assistant giving massage or special treatment should be required to hold the certificate of either the Incorporated Society of Trained Masseuses, a public hospital, or a teaching school approved by the British Medical Council. It will, the Council recognises, probably be necessary at first to make an exception in those cases in which a person by long experience may be deemed to have acquired the necessary knowledge and training.

### THE GARRETT ANDERSON MEMORIAL.

The completion of the scheme for a memorial from English women to Dr. Elizabeth Garrett Anderson by the endowment of fifty beds, is virtually complete. At the end of last week only £5,000 more was required, and there is an important fact to be added. The not too convenient site in Euston Road has been improved by a gift of land from Sir Alan Anderson and Miss Aldrich Blake. This land adjoins the hospital, which is thus provided with the means of future extension. The memorial is assured of success, and its women promoters deserve praise for the short time in which they have raised the £50,000 asked for.

### HOSPITAL MIGRATION IN BATH.

The Royal United Hospital, Bath, may leave its historic quarters if the present removal scheme is carried out. The plan is to confine the present building to a casualty station in the midst of the old quarter of the city; to sell the parts not so required; and to move the main institution to Combe Park, Weston, where the war hospital was opened in 1916. The new institution, it is suggested, shall include a pay block or wards in addition to those for free treatment. There is much to be said for the plan; and if there is a sentiment for the old buildings, this can be satisfied by conditions which will not be hard to draft if there is the will to improve them.

### THE BRITISH HOSPITAL IN PARIS.

In days when we are all travellers, English hospitals abroad have fresh interest since we may be in their neighbourhood at any time. Yet how many realise the work of the British Hospital in Paris, in the Rue de Villiers, at the Levallois Perret suburb, whose doors are open not merely to the British Colony, but to fellow-countrymen passing through Paris. The hospital, which was founded by Sir Richard Wallace, is exactly forty years old, but it needs further endowment, in fact, at least another £1,000 a year. Hospital men when in Paris should make a point of visiting the hospital, and might further be able to suggest a means of interesting English folk in the hospital, which mainly looks to the British Colony for support. The French and Italian hospitals in London are known to many of us, and the British Hospital in Paris is a valuable link with our friends, the French, in whose capital many of us like to spend part at least of our annual holiday.

### THE GROWTH OF LOCAL HOSPITALS.

The need for local hospitals, perhaps most severely felt in Wales, is general in the Midlands, and therefore the extension of Ilkeston Hospital, whereby it develops from a casualty accident station into a hospital with a surgical ward for 20 beds, a medical board of 10 beds, a private ward and a dispensary, is really welcome. The work, for which Mr. W. H. Hunt is the architect, has advanced far enough for an opening ceremony; all but £1,500 of the £10,000 required has been raised; and the administrative block should be ready in about six months or a little later. Mr. Miller Munday, of Shipley, has promised further land when required, and the effect of the new institution will be to provide promptly for local cases and thus to relieve to the extent of its capacity the waiting-list of Nottingham Hospital and of Derby Infirmary.

### THE NEW ROTHERHAM SANATORIUM.

The Rotherham Borough Council, Yorkshire, has adopted a scheme for the conversion of Oakwood Hall into a sanatorium. The scheme provides three new ward blocks with one hundred beds, and will adapt the house for an administrative block, where the recreation rooms, kitchen, and dining-rooms will be placed. The cost of the scheme is expected to be £32,000. The plan now awaits the approval of the Ministry of Health, and it will be interesting to see whether the Ministry's approval is merely formal or if the conditions of approval represent a policy of standardisation with an eye on something more than the builder's work.

### THIS WEEK'S DRUG MARKET.

Prices are well maintained on the whole and a fair amount of buying is going on. Eucalyptus oil seems to be worth attention; values have an upward tendency and there is a good demand in view of possible winter requirements. Menthol and camphor continue to move up in price, and the same can be said of cloves and clove oil. Tannic acid and gallic acid have an upward price tendency. There is a fair supply of Belgian chamomiles, and prices have a slightly lower tendency. The value of honey is firmly maintained, notwithstanding that stocks are fairly large. The scarcity of asafœtida is unrelieved. Buchu leaves are scarce. Gentian root is a trifle dearer. There is a good inquiry for jalap but very little to be had. Cassia oil and citronella oil are dearer. Olive oil is comparatively scarce and prices are higher. The supply of liquid paraffin is improving and prices have rather a lower tendency. A small arrival of rhubarb was readily snapped up, leaving prices as high as ever. Lemon oil is again dearer because of the large demand in the primary market. High prices are wanted for senna leaves.

